# Predicting grade school scientific literacy from aspects of the early home science environment

**DOI:** 10.3389/fpsyg.2023.1113196

**Published:** 2023-04-17

**Authors:** Jihye Bae, Margaret Shavlik, Christine E. Shatrowsky, Catherine A. Haden, Amy E. Booth

**Affiliations:** ^1^Little Learners Lab, Department of Psychology and Human Development, Vanderbilt University–Peabody College, Nashville, TN, United States; ^2^Children’s Memory and Learning Lab, Department of Psychology, Loyola University Chicago, Chicago, IL, United States

**Keywords:** scientific literacy, preschool science, informal STEM learning, home science environment, parent causal talk

## Abstract

Fostering scientific literacy has become an increasingly salient goal as evidence accumulates regarding the early emergence of foundational skills and knowledge in this domain, as well as their relation to long-term success and engagement. Despite the potential that the home context has for nurturing early scientific literacy, research specifying its role has been limited. In this longitudinal study, we examined associations between children’s early science-related experiences at home and their subsequent scientific literacy. Following on our previous work, we specifically considered parent causal-explanatory talk, as well as the degree to which parents facilitate access to science-related materials and experiences. A group of 153 children from diverse backgrounds were evaluated across 5 annual waves of data collection from preschool entry (*M*_age_ = 3.41) through first grade (*M*_age_ = 7.92). Results demonstrate that parent invitations for children to explain causal phenomena had strong concurrent relations to scientific literacy but showed little relation to subsequent literacy. In contrast, the broader home science environment at preschool entry, particularly in the form of exposure to science-related activities, predicted scientific literacy over the next 4 years. The directionality and specificity of these relations were clarified through the inclusion of measures of cognitive and broader home experiences as controls in regression analyses. Overall, our investigation revealed that exposure to science-related input provided by parents has particularly powerful potential for shaping scientific literacy when children are very young. Implications for parent-focused interventions that promote science literacy are discussed.

## 1. Introduction

Given the importance of STEM literacy for personal and societal health and success, considerable discourse and empirical investigation has focused on better equipping children to thrive in these fields. Although the majority of this work has centered around school-aged children or typically children 5 years and up in the USA (e.g., Bathgate et al., 2014; Sha et al., 2016), researchers have increasingly recognized the importance of studying the earliest origins of scientific literacy in younger children (e.g., Alexander et al., 2012; Leibham et al., 2013).

Scientific literacy refers to our understanding of core disciplinary ideas (e.g., physics) and practices (e.g., defining problems, interpreting data), as well as cross cutting concepts (e.g., cause and effect, patterns) as reflected in the current Next Generation Science Standards (NGSS Lead States, 2013). It is now clear that scientific literacy emerges early (Eshach, 2006; Duschl et al., 2007) and that individual and group-level variability in related knowledge and skills is evident prior to school-entry (National Science Board, 2019). Moreover, early scientific knowledge is predictive of success in science throughout grade school (Morgan et al., 2016; Byrnes et al., 2018; Kähler et al., 2020). Better understanding the foundations of scientific literacy is therefore particularly important for discovering ways to support long-term engagement and success in science.

Although the development of evidence-based preschool science curricula (e.g., Peterson and French, 2008; Gelman et al., 2010; Gonzalez et al., 2010) has been an important part of efforts to launch children on positive developmental trajectories in STEM, informal learning contexts have also been highlighted as providing foundational experiences (e.g., Vandermaas-Peeler et al., 2016, 2019; Willard et al., 2019). Of these, the home environment might be particularly impactful in nurturing scientific literacy (e.g., Dearing and Tang, 2010; Schaub, 2015). The current study focuses specifically on parents as a dominant force in shaping the early home environment and a source of experiences potentially relevant to the early emergence and development of children’s scientific literacy (Bandura and Walters, 1963; Jacobs and Eccles, 2000; Davis-Kean, 2005). Based on the existing literature, we consider two distinct ways in which parents might exert their influence.

First, we consider the degree to which parents evoke causal explanations in conversations with their children. Causal explanations are descriptions of how or why factors in a system influence each other. When parents scaffold children’s consideration of causal explanations, it may support scientific literacy by both contributing directly to conceptual knowledge and by offering opportunities for practicing scientific inquiry processes like hypothesis generation and revision. Several studies have broadly observed the frequency and quality of conversations between parents and their children in a variety of informal science settings (Callanan and Jipson, 2001; Crowley and Galco, 2001; Haden et al., 2014; Van Schijndel and Raijmakers, 2016). For example, Crowley et al. (2001) report that young children are better able to process the causal structure of museum exhibits after exploring them with their parents (see also Willard et al., 2019). Similarly, Callanan et al. (2019) found that parent’s causal explanations supported child’s systematic exploration of museum exhibits, and that this relationship did not vary by age or gender of the child. Booth et al. (2020) more specifically found that parent’s *invitations* for their 3-year-olds to generate causal explanations during free play correlate with the children’s concurrent scientific literacy.

Second, we consider the broader home science environment, including access to science-related materials and experiences provided by parents (Westerberg et al., 2022). Empirical research has already linked other specific aspects of the early home learning environment with corresponding domain-specific achievements, such as reading literacy (Sénéchal and LeFevre, 2002; Rodriguez and Tamis-LeMonda, 2011) and math (Hart et al., 2016; Napoli and Purpura, 2018). Despite its potential for similar associations to long-term success (e.g., Bell et al., 2009), the home science environment has received much less attention (Ellis et al., 2022). In one of the few relevant studies, however, Junge et al. (2021) report that home science activities are not only associated with preschooler’s scientific knowledge, but that they mediate the association between overall home learning environment (e.g., socioeconomic status, parental interest in science), and child’s scientific knowledge (see also Vandermaas-Peeler et al., 2018). Although Booth et al. (2020) only considered the broader home science environment as a control in their analyses of causal talk, they too discovered that it accounted for unique variance in concurrent scientific literacy at 3 years of age.

This work aims to further clarify links between aspects of early experience and emergent scientific literacy by examining four years of longitudinal data beyond the initial wave reported in Booth et al. (2020).We first ask whether associations between parents’ causal talk and scientific literacy observed at 3 years of age replicate throughout early childhood. Although Booth et al. (2020) failed to find an association between parent-generated explanations and scientific literacy, we reconsider this theoretically relevant metric in order to evaluate potentially longer-term effects through first grade (7-8-year-olds). We also consider whether the association between parent invitations for their children to explain causal phenomena and scientific literacy observed in Booth et al. (2020) extends to subsequent measurements. These analyses will further clarify the links between aspects of early causally oriented conversations with caregivers and emergent scientific literacy.

We then ask whether broader indicators of the home science environment are potentially foundational to early scientific literacy. In addition to the composite measure of home science used in Booth et al. (2020), we further examine potential divergence in the effects of components thereof. Specifically, we reasoned that science-related experiences (e.g., conducting science experiments) might be more powerful in supporting the development of scientific literacy than exposure to science-related materials (e.g., science books), given that the latter are likely to be useful only to the extent that they are incorporated into the former. This might be especially true for very young children who are limited in their ability to productively explore science-related materials on their own. We suspect that the home environment will become relatively less predictive of scientific literacy as children are increasingly exposed to science in a variety of other contexts (e.g., preschool) and come to exert more control over which activities they engage in (Maccoby 1984; Bergin 2016).

Throughout, we capitalize on the longitudinal nature of the data to achieve greater precision in our conclusions. Specifically, we clarify the directionality of effects by controlling for initial scientific literacy. We also clarify the specificity of observed associations by controlling for general cognitive skills and broader (non-science) cognitive stimulation in the home, and by considering contrastive predictions to math and reading skills.

## 2. Methods

### 2.1. Participants

An *a priori* power analysis using G*Power software (Faul et al., 2007) suggested a sample size of 120–150 children for our longitudinal observational study. As part of a larger longitudinal study, 153 children were recruited from a database of families interested in research from Austin, Texas and surrounding areas (81 female, *M*_age_ = 3.41 years, SD = 0.26, range = 3.01–3.92). Child participants were described by their caregivers as proficient in English and absent of any diagnosed developmental delay or disorder. At the first session, eight additional children were excluded based on their inability to follow instructions due to inadequate English knowledge or behavioral noncompliance. The sample was demographically diverse across race, ethnicity, and maternal education (see Table 1). At the second wave of data collection, 120 (64 female, *M*_age_ = 4.59 years, SD = 0.26, range = 3.66–5.09) remained in the study, at the third wave 112 (61 female, *M*_age_ = 5.02 years, SD = 0.23, range = 5.02–5.92) remained, at the fourth wave 88 (43 female, *M*_age_ = 6.78 years, SD = 0.23, range = 6.04–7.66) remained, and at the final wave 87 (47 female, *M*_age_ = 7.92 years, SD = 0.28, range = 7.12–8.51). Throughout these years of assessment, attrition was primarily due to families moving out of town or our inability to re-establish contact. Data collection at the fourth wave was also substantially disrupted by the COVID-19 pandemic, resulting in a spike in attrition and an eventual shift to virtual data collection (see Table 2).

**Table 1:** Participant demographics (in percentages) across 5 years of data collection.

		Wave 1(*n* = 153)	Wave 2(*n* = 120)	Wave 3(*n* = 112)	Wave 4(*n* = 88)	Wave 5(*n* = 87)
Race	White/Caucasian	73.9	74.2	78.4	78.4	80.5
	Black/African American	13.1	12.5	7.2	4.5	4.6
	Asian/Asian American	2.6	1.7	0.9	2.3	2.3
	Mixed Race/Other	10.5	11.7	13.5	14.8	12.6
Ethnicity	Non-Hispanic/Latino	69.9	72.5	68.5	70.5	70.1
	Hispanic/Latino	30.1	27.5	31.5	29.5	29.9
Maternal Education	No more than high school	27.5	20.9	18.0	12.5	14.9
	Technical or Associate’s degree	6.5	6.7	6.3	6.8	3.4
	Bachelor’s degree	38.6	44.2	46.8	46.6	49.4
	Master’s degree	18.9	20.0	19.8	22.7	24.1
	Advanced degree	8.5	8.3	9.0	11.4	8.0

**Table 2:** Missing data (in percentages) and reasons for missingness.


Task	% Missing	Top 3 Reasons for Missingness (*n*)^†^
Wave 1	Causal Talk	17.65	Technical (*n* = 17), Attrition (*n* = 10)
	Scientific Literacy-Lens	3.27	Attrition (*n* = 4), Behavioral (*n* = 1)
	Home Science	3.92	Incomplete (*n* = 6)
	StimQ-P	18.30	Attrition (*n* = 27), Incomplete (*n* = 1)
	Causal Talk	25.49	Attrition (*n* = 39)
	NIH-ECB		
	PVT	1.31	Attrition (*n* = 1), Behavioral (*n* = 1)
	FL	24.18	Attrition (*n* = 24), Failed Training (*n* = 7), Behavioral (*n* = 5)	DCCS	32.68	Attrition (*n* = 29), Failed Training (*n* = 11), Behavioral (*n* = 8)
	PS	39.47	Attrition (*n* = 28), Failed Training (*n* = 15), Technical (*n* = 15)	Wave 2
	Scientific Literacy-Lens	26.97	Attrition (*n* = 33), Behavioral (*n* = 7)
Home Science	22.88	Attrition (*n* = 34), Incomplete (*n* = 1)
NIH-ECB		
PVT	21.57	Attrition (*n* = 33)
FL	28.10	Attrition (*n* = 40), Behavioral (*n* = 2), Failed Training (*n* = 1)
DCCS	31.37	Attrition (*n* = 42), Behavioral (*n* = 5), Failed Training (*n* = 1)
PS	35.29	Attrition (*n* = 42), Failed Training (*n* = 9), Behavioral (*n* = 3)
Wave 3	Causal Talk	32.68	Attrition (*n* = 46), Technical (*n* = 4)
	Scientific Literacy-Lens	34.64	Attrition (*n* = 41), Behavioral (*n* = 8), Technical (*n* = 4)
	Scientific Literacy-SLA	36.60	Attrition (*n* = 56)
	Home Science	46.05	Attrition (*n* = 50), Incomplete (*n* = 20), Experimenter Error (*n* = 1)
	Wave 4	Scientific Literacy-SLA	49.67	Attrition (*n* = 74), Behavioral (*n* = 2)
Scientific Literacy-TNSci		43.79	Attrition (*n* = 65), Incomplete (*n* = 2)
Home Science		50.33	Attrition (*n* = 76), Incomplete (*n* = 1)
Wave 5		Scientific Literacy-SKI	50.34	Attrition (*n* = 75)
	Scientific Literacy-TNSci	44.74	Attrition (*n* = 65), Behavioral (*n* = 2), Incomplete (*n* = 1)
	Home Science	58.82	Attrition (*n* = 80), Incomplete (*n* = 10)
	TNMath	60.53	Attrition (*n* = 78), Incomplete (*n* = 13), Behavioral (*n* = 1)
	NIH TORRT	50.98	Attrition (*n* = 77), Incomplete (*n* = 1)

NIH-ECB = NIH Toolbox Early Cognition Battery; PVT = Picture Vocabulary Test; FL = Flanker Inhibitory Control and Attention Test; DCCS = Dimensional Change Card Sort Test; PS = Picture Sequence Memory Test; StimQ-P = StimQ-Preschool; Lens = Lens on Science; SLA = Science Learning Assessment; SKI = Science Knowledge Inventory; NIH TORRT = NIH Toolbox Oral Reading Recognition Test; TNSci = TerraNova Science; TNMath = TerraNova Math; Home Science = Home Science Environment.^†^Total *N* = 153. Attrition within a given wave of data collection was possible given that tasks were administered across multiple sessions.

### 2.2. Procedure

One wave of data was collected each year, for 5 years, and each wave included between two and five sessions of testing. Sessions were video-recorded and later coded offline. Each session included between three to six tasks (always presented in the same order) and lasted between 30 and 60 min. All Wave 1, 2, and 3 sessions were conducted in a laboratory setting except for the very first session of Wave 1 which was conducted at a local science museum. Although testing began in the laboratory for Wave 4, the global pandemic necessitated that we shift to virtual format. Wave 5 was also conducted virtually. After each session, caregivers received financial compensation and, if the session was run in person, the child was also given a book.

### 2.3. Measures

Our investigation included measures of parent causal talk and the broader home science environment, as well as children’s scientific literacy and general cognitive ability. See Table 3 for measures used at each wave.

**Table 3:** Measures used at each timepoint of data collection.

Construct	Measure	Wave 1	Wave 2	Wave 3	Wave 4	Wave 5
Parent input	Parent Causal Talk	✓	✓	✓		
Broader home environment	Home Science Environment Survey	✓	✓	✓	✓	✓
Scientific literacy	Lens on Science	✓	✓	✓		
	SLA			✓	✓	
	TerraNova Science				✓	✓
	SKI					✓
Cognitive simulation and ability	StimQ-Preschool	✓				
	NIH-ECB	✓	✓			
	TerraNova Math					✓
	NIH TORRT					✓

SLA, Science Learning Assessment; SKI, Science Knowledge Inventory; NIH-ECB, NIH Toolbox Early Cognition Battery; NIH TORRT, NIH Toolbox Oral Reading Recognition Test.

#### 2.3.1. Home environment

##### 2.3.1.1. Parent causal talk

Parent–child dyads played freely with toys affording causal explanations at Waves 1 (sink-float task in the lab and a launcher museum exhibit that involved building and testing airplanes), 2 (Hasbro’s Mouse Trap™ game) and 3 (balance scale task). Based on pilot observations and timing constraints, participants were allotted 10 min to play with each set of toys. They were given no specific instructions about how to interact with the toys and were only told to let the experimenter know if they wanted to stop early. The 10 min of play were coded offline and broken into 60 s windows. For each window, coders indicated if the parent (1) produced a causal explanation, (2) invited the child to explain a causal phenomenon, and (3) provided any other causally relevant utterance. Utterances coded as causal explanations often included “because” or “if, then” statements, such as, “If I put the ball here, then this will make it fall.” Likewise, utterances coded as causal invitations often contained “why” or “how,” such as, “Why do you think that one sank?.” See Table 4 for more examples of utterances and how they were coded.

**Table 4:** Parent causal talk: example utterances for code types.

Code type	Example utterances
Causal explanations	This one is heavier so that’s why it goes to the bottom. The higher the pressure is, the further it will fly. I think that tail made it a little heavy. It did not go quite as far, huh?
Causal invitations	Why do you think they sink to the bottom? Why is it floating? How are you going to make it fly?
(Other causal talk)	Which ones do you think will sink? What happens if you put it that way? The ones on the bottom are heaviest. Were you adjusting the little knob over there before? You think we should try a smaller tail? Is it aimed okay? That one sinks. See, it goes straight to the bottom.

For each of these causal constructs, the proportion of 60 s windows (out of the total maximum of 10) in which parents produced at least one utterance of each target construct was calculated. Utterances were coded as causally oriented even if the information provided in that utterance was not technically correct (e.g., referencing size instead of density/weight). The proportion of windows in which parents specifically produced an explanation or an invitation served as the key dependent variables. Note that because two play sessions were implemented at Wave 1 (in the lab and museum), final proportions were attained by averaging across contexts. An additional research assistant independently coded 20 percent of the co-play sessions. Although reliability was generally good (all inter-class correlations >0.75), discrepancies were jointly reviewed to arrive at full consensus.

##### 2.3.1.2. Home science environment

A parent survey was administered at each wave of data collection asking about the number of science-themed books, toys, and apps/computer games available in the home. No specific examples were provided for these home resources and it was left to the caregiver to determine what counted as “science.” These responses were each coded into bins (1–5 = 1, 6–20 = 2, 21–50 = 3, and 50+ = 4) and then summed across types to calculate a ‘materials’ score ranging from 0 to 12. Parents were also asked about the frequency (on a 7-point scale with 0 = never and 6 = almost every day) with which they participate in science activities (e.g., like reading science books and conducting experiments) with their child, as well as how often they visit science fairs or museums with their children (0 = never, 6 = every week or two). These were then summed into an ‘activities’ score ranging from 0 to 12. The material and activities scores were then summed into the total home science environment score ranging from 0 to 24. See Meyer (1990) and Jacobs and Bleeker (2004) for similar assessments.

##### 2.3.1.3. Cognitive stimulation

The StimQ-Preschool (Mendelsohn et al., 1999) scale measures cognitive stimulation present in the home environment of children ages 3 to 6. Questions focus on the availability of learning materials, frequency of reading, parental involvement, and parent verbal responsivity with scores ranging from 0 to 89. This measure was not included in the design of the original longitudinal study but was collected on the same sample at Wave 1 for a related student project. It is included here as a useful control in assessing the specificity of effects.

#### 2.3.2. Scientific literacy

Different developmentally appropriate measures of scientific literacy were utilized at each measurement time point. More information on the scientific literacy measures is available on OSF.^1^

##### 2.3.2.1. Lens on science

Lens on Science (Greenfield, 2015) is an adaptive computerized measure developed for children aged 3 to 5 that aims to assess all the scientific literacy components specified in the U.S. national science education guidelines (National Research Council, 2012). It takes approximately 15 min to administer, during which 35 to 40 items are presented (from a bank of 498 items). The items represent the three broad domains of life, earth and space, and physical and energy science, as well as eight core science practices (observing, describing, comparing, questioning, predicting, experimenting, reflecting, and cooperating). Children are instructed to respond to an item by selecting one of several images on a tablet touchscreen. Upon completion, each child received a standard item response theory (IRT) ability score ranging from −3 to 3. High reliability of 0.87 is reported by Greenfield (2015).

##### 2.3.2.2. Science learning assessment (SLA)

The SLA (Samarapungaven et al., 2009) is designed for kindergarten students kindergarten students (ages 5 and 6) and consists of 24 items broken into two subtests: the Scientific Inquiry Processes subtest and the Live Science Concepts subtest. The Scientific Inquiry Processes subtest asks about children’s understanding of how science is conducted (e.g., making predictions, understanding simple scientific tools) and children select among three possible answers presented visually and verbally. The Life Science Concepts subtest asks about children’s knowledge of living things and the physical world in multiple choice format (e.g., choosing the correct name of an animal that corresponds to the picture shown) and in free response questions (e.g., mechanism in which insects move). Scores range from 0 to 38. Internal reliability is reported as 0.79 by Samarapungavan et al. (2009).

##### 2.3.2.3. TerraNova science subtest (third edition)

TerraNova Science Subtest (CTB/McGraw-Hill LLC, 2010) is a standardized norm-referenced achievement test that taps into knowledge in core science content areas (life, earth, physical science, and scientific inquiry) for students in grade school (K-12 in the U.S.). The test consists of 20 multiple choice questions and its raw scores range from 0 to 20.

##### 2.3.2.4. Science knowledge inventory (SKI)

Administered at the last wave, the SKI (Koerber and Osterhaus, 2019) consists of 30 multiple-choice items equally drawn from three areas: experimentation, data interpretation, and understanding the nature of science (e.g., what scientists do, what scientists ask). For each item, the experimenter reads a brief description of a character who wishes to find something out about science. Children choose their answers to each item from among three illustrated options. Internal reliability of 0.78 is reported by Koerber and Osterhaus (2019). Full measure is available on the original authors’ OSF.^2^

#### 2.3.3. Cognitive ability

##### 2.3.3.1. NIH toolbox early childhood cognition battery (ECB)

The ECB (Bauer and Zelazo, 2014) is a highly reliable measure of children’s overall cognitive functioning, consisting of four tasks: the Dimension Change Card Sort Test, (assessing cognitive flexibility), the Flanker Inhibitory Control and Attention Test (assessing inhibitory control), the Picture Sequence Memory Test (assessing episodic memory), and the Picture Vocabulary Test (assessing receptive vocabulary). Composite age-adjusted scaled scores (with a mean of 100) were calculated after imputing values for missing component tasks. This task was used to control for general cognitive abilities in regression analyses.

##### 2.3.3.2. TerraNova math subtest (third edition)

As with TerraNova Science Subtest, the Math Subtest (CTB/McGraw-Hill LLC, 2010) is a norm-standardized achievement test of math ability that includes 47 questions. This test was used as a contrast case in specifying the precision of associations to scientific literacy in our analyses. Raw scores range from 0 to 47.

##### 2.3.3.3. NIH toolbox oral reading recognition test (TORRT)

The TORTT (Gershon et al., 2013) is an adaptive test that measures children’s pronunciation of individual printed words and naming and recognition of individual printed letters presented on a tablet.” Raw scores range from 0 to 20. This test was also used as a contrast case in specifying the precision of associations to scientific literacy in our analyses.

## 3. Results

We first evaluated whether missing data caused systematic variability across key demographic factors or measurements. Little’s Test (Little, 1988) was not significant, suggesting that our data were missing completely at random (MCAR), *χ*^2^ (3441) = 3450.59, *p* = 0.404. To address missing data in a maximally unbiased manner, we conducted 100 iterations of multiple imputation including our home science environment and scientific literacy measures. Demographic and cognitive variables that correlated highly (*r* > 0.40) with our key measures (and had no more than 25% missingness themselves) were also included as auxiliary variables in the imputation (see Johnson and Young, 2011). Child gender was included in these preliminary analyses of demographic variables and did not correlate with any of our key variables, including parent causal talk, and hence was not included in the current analysis.

After imputation, we combined our scientific literacy scores into a single composite score for waves where multiple measures were available (i.e., Waves 3, 4, and 5). This decision was based on the face validity of conceptual equivalence across our scientific literacy measures and the significant correlations between them at each measurement wave (see Table 5). Next, we conduct a series of bivariate correlation and multiple regression analyses to investigate our major research questions.

**Table 5:** Correlations for scientific literacy measures at Wave 3, 4, and 5.

		1	2	3	4	5	6
Scientific literacy	1. Lens3	–					
	2. SLA3	0.46**	–				
	3. SLA4	0.38**	0.49**	–			
	4. TNSci4	0.35*	0.36**	0.47**	–		
	5. SKI	0.41*	0.45**	0.42*	0.37*	–	
	6. TNSci5	0.38*	0.43**	0.44**	0.55**	0.32*	–

Lens, Lens on Science; SLA, Science Learning Assessment; TNSci, TerraNova Science Subtest; SKI, Science Knowledge Inventory. The number following the task abbreviation is the measurement wave. **p < 0.01, *p < 0.05.

### 3.1. Does parent causal talk relate to scientific literacy?

As a first step, we examined descriptive statistics and bivariate correlations between parent causal talk and scientific literacy measures. As expected, and consistent with Booth et al. (2020), parent invitations for children to explain causal phenomena at Wave 1 had a significant positive association with children’s concurrent scientific literacy (*r* = 0.24*, p =* 0.006). Although parent invitations to explain at Waves 2 and 3 failed to correlate with either concurrent or subsequent scientific literacy, parent invitations to explain at Wave 1 did also correlate with scientific literacy one year later (*r* = 0.24*, p =* 0.012). In contrast, parent-produced causal explanations failed to correlate with scientific literacy in any analysis (see Table 6), which is also consistent with Booth et al. (2020).

**Table 6:** Bivariate correlations for parent talk and scientific literacy.

		Parent causal talk						*M (SD)*
		Wave 1		Wave 2		Wave 3		
		Exp	Invite	Exp	Invite	Exp	Invite	
Scientific Literacy^†^	Wave 1	0.028	0.24*	0.05	0.11	−0.05	0.00	0.36 (1.00)
	Wave 2	−0.03	0.24*	0.020	0.12	−0.06	0.15	1.51 (1.00)
	Wave 3	−0.01	0.16	0.16	0.20	0.05	0.20	−0.14 (0.97)
	Wave 4	−0.01	0.14	0.09	0.18	0.08	0.11	−0.22 (1.06)
	Wave 5	−0.04	0.08	0.02	0.10	0.04	0.15	−0.01 (1.00)
	*M* (SD)	0.13 (0.13)	0.09 (0.11)	0.17 (0.17)	0.08 (0.15)	0.14 (0.17)	0.15 (0.13)	

Exp, Casual Explanations; Invite, Invitations to Explain Causal Phenomenon. ^†^Scientific Literacy at Wave 3, 4, and 5 is a standardized composite score but is a single score at Wave 1 and Wave 2. **p < 0.01, *p < 0.05.

Given that (1) parent invitations-to-explain correlated with scientific literacy at Wave 1 and (2) scientific literacy was stable across Wave 1 and 2, it was important to clarify whether the observed association between parent invitations-to-explain at Wave 1 and scientific literacy at Wave 2 was truly predictive (rather than merely a spurious byproduct of these other two associations). We therefore conducted a multiple regression analysis, controlling for baseline (Wave 1) scientific literacy scores. Consistent with an early association carried forward by stability in measurement of scientific literacy, parent invitations were no longer predictive of scientific literacy scores at Wave 2 in this analysis, *B* = 0.801 (SE = 0.718), *p* = 0.265.

### 3.2. Does the home science environment relate to scientific literacy?

In parallel with our analyses of aspects of parent causal talk, our first step in investigating relations between home environment and scientific literacy was to calculate descriptive statistics and bivariate correlations between home science environment and scientific literacy scores. We observed significant associations between home science environment at Wave 1 (*r* = 0.18, *p* = 0.028) and Wave 2 (*r* = 0.30, *p* < 0.001), and Wave 3 (*r* = 0.31, *p* < 0.001) scientific literacy scores. The home science environment at Wave 2 was significantly positively related to scientific literacy at all waves, with the strongest associations observed at Wave 4 (*r* = 0.34, *p* = 0.003) and Wave 5 (*r* = 0.32, *p* = 0.004). Although later home science environment scores were less strongly correlated with scientific literacy scores in general, science environment scores at Waves 3, 4, and 5 were significantly associated with scientific literacy at Wave 5 (see Table 7).

**Table 7:** Bivariate correlations for home science environment and scientific literacy.

		Home Science Environment				
		Wave 1	Wave 2	Wave 3	Wave 4	Wave 5
Scientific literacy^†^	Wave 1	0.18*	0.23*	0.11	0.22*	0.13
	Wave 2	0.30**	0.26*	0.18	0.25*	0.19
	Wave 3	0.31**	0.23*	0.20	0.24	0.19
	Wave 4	0.19	0.34**	0.20	0.38*	0.29
	Wave 5	0.15	0.32**	0.31*	0.33*	0.37*	*M (SD)*	12.33 (3.72)	13.94 (3.90)	14.18 (3.76)	12.76 (4.21)	13.42 (5.08)

^†^Scientific Literacy at Wave 3, 4, and 5 is a standardized composite score but is a single score at Wave 1 and Wave 2. **p < 0.01; *p < 0.05.

We next evaluated whether significant correlations observed across waves of data collection truly reflected predictive relations (as opposed to residual effects of the relative stability of scientific literacy). To this end, we first ran a multivariate regression analysis with home science environment at Wave 1 predicting scientific literacy at Waves 1, 2, and 3, while controlling for baseline (Wave 1) scientific literacy (see Table 8, Model 1). Wave 1 home science environment continued to predict subsequent scientific literacy scores at Wave 2, *B* = 0.05 (SE = 0.02), *p* < 0.01 and Wave 3, *B* = 0.06 (SE = 0.10), *p* < 0.01, over and above baseline scientific literacy. To explore the possibility that the observed relation between home science environment and subsequent scientific literacy was due to a common reliance on broad cognitive skills, we ran a follow-up regression analysis including NIH-ECB as an additional predictor. Indeed, when controlling for baseline cognitive skills, the Wave 1 home science environment no longer accounted for a significant amount of variance in Wave 2 scientific literacy scores, *B* = 0.04 (SE = 0.02), *p* = *0*.076, although a trend was still evident. The home science environment did, however, predict Wave 3 scientific literacy scores, *B* = 0.05 (SE = 0.02), *p* < *0*.05 over and above baseline cognitive skills (see Table 8, Model 2).

**Table 8:** Multivariate regression analyses for home science environment at Wave 1 predicting scientific literacy at Wave 2 and 3.

Outcome	Predictor	Unstandardized		Standardized	*t*	*p*	95% CI		*sr*
		*B*	SE	β			*LL*	*UL*	
**Model 1: Controlling for baseline scientific literacy**									
SciLit2^†^	Intercept	0.71	0.25	−0.06	2.82	0.005	0.22	1.20	
	HomeSci1	0.05	0.02	0.18	2.58	0.010	0.01	0.09	0.19
	Lens1	0.62	0.01	0.64	8.05	<0.001	0.47	0.77	0.61
SciLit3	Intercept	−1.04	0.70	0.02	−3.66	<0.001	−1.60	−0.48	
	HomeSci1	0.06	0.10	0.26	2.68	0.007	0.02	0.10	0.23
	Lens1	0.44	0.01	0.43	5.39	<0.001	0.28	0.60	0.45
**Model 2: Controlling for baseline scientific literacy and cognitive skills**									
SciLit2	Intercept	−0.58	0.55	−0.07	−1.06	0.291	−1.65	0.49	
	HomeSci1	0.04	0.20	0.12	1.78	0.076	0.00	0.08	0.13
	Lens1	0.51	0.09	0.52	5.85	<0.001	0.34	0.69	0.43
	NIH-ECB1	0.02	0.01	0.22	2.64	0.008	0.00	0.03	0.17
SciLit3	Intercept	−1.89	0.63	0.01	−2.89	0.004	−3.17	−0.61	
	HomeSci1	0.05	0.02	0.21	2.14	0.032	0.00	0.10	0.18
	Lens1	0.37	0.09	0.33	3.93	<0.001	0.18	0.55	0.32
	NIH-ECB1	0.01	0.01	0.18	1.46	0.145	0.00	0.02	0.12

SciLit, Scientific Literacy; Lens, Lens on Science; HomeSci, Home Science Environment; NIH-ECB, NIH Toolbox Early Childhood Cognition Battery; CI, confidence interval; LL, lower limit; UL, upper limit; sr, semi-partial (part) correlation. The number following the task abbreviation is the measurement wave. ^†^SciLit at Wave 3 is a standardized composite score but is a single score at Wave 2.

We then turned our attention to the relation between Wave 2 home science environment and subsequent scientific literacy scores. Again, we added baseline scientific literacy score as a predictor in the regression analysis (see Table 9, Model 1) and followed up with adding baseline cognitive skills as an additional predictor (Table 9, Model 2). When controlling for Wave 2 scientific literacy scores, we see Wave 2 home science environment predicting Wave 4 scientific literacy scores, *B* = 0.06 (SE = 0.03), *p* < *0*.05, and trending toward significance for Wave 5 but not at Wave 3. When Wave 2 cognitive skills are added into the regression, the Wave 2 home science environment no longer accounts for significant variance in any subsequent scientific literacy score, although trends remain near significance for Waves 4 and 5.

**Table 9:** Multivariate regression analyses for home science experience at Wave 2 predicting scientific literacy at Waves 3, 4, and 5.

Outcome	Predictor	Unstandardized		Standardized	*t*	*p*	95% CI		*sr*
		*B*	SE	β			*LL*	*UL*	
**Model 1: Controlling for baseline scientific literacy**									
SciLit3	Intercept	−1.31	0.33	0.08	−3.95	<0.001	−1.96	−0.66	
	HomeSci2	0.02	0.02	0.10	0.76	0.446	−0.03	0.06	0.07
	Lens2	0.59	0.09	0.59	6.70	<0.001	0.42	0.77	0.59
SciLit4	Intercept	−1.86	0.42	0.11	−4.44	<0.001	−2.68	−1.03	
	HomeSci2	0.06	0.03	0.21	2.00	0.046	0.00	0.11	0.20
	Lens2	0.55	0.11	0.49	4.91	<0.001	0.33	0.77	0.51
SciLit5	Intercept	−1.53	0.39	0.08	−3.91	<0.001	−2.30	−0.76	
	HomeSci2	0.05	0.03	0.14	1.87	0.063	0.00	0.10	0.18
	Lens2	0.53	0.11	0.51	4.97	<0.001	0.33	0.74	0.52
**Model 2: Controlling for baseline scientific literacy and cognitive skills**									
SciLit3	Intercept	−2.24	0.69	0.06	−3.26	<0.001	−3.58	−0.89	
	HomeSci2	0.02	0.02	0.09	0.71	0.481	−0.03	0.06	0.06
	Lens2	0.52	0.11	0.51	4.93	<0.001	0.31	0.72	0.43
	NIH-ECB2	0.01	0.01	0.13	1.51	0.131	0.00	0.02	0.12
SciLit4	Intercept	−2.90	0.82	0.10	−3.55	<0.001	−4.51	−1.30	
	HomeSci2	0.05	0.03	0.20	1.94	0.054	0.00	0.11	0.19
	Lens2	0.47	0.14	0.40	3.41	<0.001	0.20	0.73	0.36
	NIH-ECB2	0.01	0.01	0.15	1.37	0.172	−0.01	0.03	0.13
SciLit5	Intercept	−2.09	0.77	0.07	−2.70	0.007	−3.60	−0.57	
	HomeSci2	0.05	0.03	0.13	1.81	0.072	0.00	0.09	0.17
	Lens2	0.48	0.12	0.45	3.88	<0.001	0.24	0.73	0.40
	NIH-ECB42	0.01	0.01	0.09	0.08	0.422	−0.01	0.02	0.07

SciLit, Scientific Literacy (composite score); Lens, Lens on Science; HomeSci, Home Science Environment; NIH-ECB, NIH toolbox Early Childhood Cognition Battery; CI, confidence interval; LL, lower limit; UL, upper limit; sr, semi-partial (part) correlation. The number following the task abbreviation is the measurement wave.

Although the analyses reported thus far are consistent with at least some truly predictive relations between the early home science environment and subsequent scientific literacy, it is still possible that the reciprocal relation exists whereby a child’s level of scientific literacy might shape their subsequent home literacy environment. Some significant bivariate correlations seen in Table 7 were consistent with this possibility. Specifically, Wave 1 scientific literacy was positively correlated with the home science environment at Waves 2 and 4 (see Table 7). Also, Wave 2 scientific literacy was correlated with home science environment at Wave 4, and Wave 4 scientific literacy was correlated with home science at Wave 5. To clarify the nature of these associations, we ran additional regression analysis with home science environment as the outcome and scientific literacy as the predictor, controlling for baseline home science environment. None of the observed reciprocal relations held under these circumstances (all *ps* > 0.10).

In further exploratory analyses, we examined the materials and activities subcomponents of the home science environment score separately. As can be seen in Table 10, significant bivariate correlations were evident between science *activities* at Wave 1 and scientific literacy measured concurrently at all subsequent waves. Science *materials*, in contrast, only correlated relatively weakly with scientific literacy at the second wave of measurement. These associations reversed somewhat when the home science environment at Wave 2 was instead considered. Here, science activities only correlated significantly with scientific literacy at Wave 4 and 5 while science materials correlated significantly with all waves of scientific literacy, although only at a trend level for Wave 3. Correlations between later measures of home science activities and materials with scientific literacy were only sporadically observed.

**Table 10:** Bivariate correlations for home science activities, home science materials, and scientific literacy.

		Home science environment									
		Wave 1		Wave 2		Wave 3		Wave 4		Wave 5	
		Act	Mat	Act	Mat	Act	Mat	Act	Mat	Act	Mat
Scientific Literacy^†^	Wave 1	0.18*	0.11	0.13	0.22*	0.00	0.17	0.13	0.18	0.05	0.15
	Wave 2	0.30**	0.19*	0.18	0.23*	0.03	0.25*	0.14	0.21	0.06	0.23
	Wave 3	0.37**	0.15.	0.15	0.21	0.16	0.17	0.33*	0.06	0.19	0.12
	Wave 4	0.29**	0.02	0.28*	0.27*	0.17	0.15	0.25	0.23	0.22	0.24
	Wave 5	0.26*	−0.01	0.26*	0.26*	0.21	0.27*	0.23	0.25	0.22	0.35*

Act, Activity; Mat, Material. ^†^Scientific Literacy at Wave 3, 4, and 5 is a standardized composite score but is a single score at Wave 1 and Wave 2. **p < 0.01, *p < 0.05.

Importantly, when submitted to regression analyses including baseline scientific literacy (see Table 11, Model 1), all associations between science activities at 3 years and subsequent scientific literacy held (albeit at only a trend level for Wave 5). Associations further weaken when cognitive skill is added as a control (see Table 11, Model 2), although scientific activities at 3 years still account for significant variance in scientific literacy at Wave 3, and trend toward doing so at Wave 2 as well.

**Table 11:** Multivariate regression analyses for home science activity at Wave 1 predicting scientific literacy at Waves 2, 3, 4, and 5.

Outcome	Predictor	Unstandardized		Standardized	*t*	*p*	95% CI		*sr*
		*B*	SE	*β*			LL	UL	
**Model 1: Controlling for baseline scientific literacy**									
SciLit2^†^	Intercept	0.71	0.24	−0.05	3.01	0.00	0.25	1.18	
	HomeAct1	0.08	0.03	0.19	2.71	0.01	0.02	0.14	0.19
	Lens1	0.62	0.08	0.64	8.06	<0.001	0.47	0.77	0.61
SciLit3	Intercept	−1.20	0.27	0.03	−4.45	<0.001	−1.73	−0.67	
	HomeAct1	0.121.	0.03	0.29	3.51	<0.001	0.05	0.19	0.28
	Lens1	0.43	0.08	0.42	5.37	<0.001	0.27	0.59	0.44
SciLit4	Intercept	−1.12	0.34	0.08	−3.26	0.00	−1.79	−0.44	
	HomeAct1	0.10	0.04	0.22	2.27	0.02	0.01	0.18	0.21
	Lens1	0.48	0.11	0.41	4.61	<0.001	0.28	0.69	0.45
SciLit5	Intercept	−0.80	0.34	0.04	−2.35	0.02	−1.48	−0.13	
	HomeAct1	0.08	0.04	0.15	1.91	0.06	0.00	0.16	0.18
	Lens1	0.38	0.10	0.35	3.81	<0.001	0.18	0.57	0.38
**Model 2: Controlling for baseline scientific literacy and cognitive skills**									
SciLit2	Intercept	−1.03	0.74	−0.07	−1.39	0.17	−2.49	0.43	
	HomeAct1	0.04	0.04	0.10	1.24	0.22	−0.03	0.11	0.08
	Lens1	0.50	0.09	0.51	5.57	<0.001	0.32	0.67	0.41
	NIH-ECB1	0.01	0.01	0.20	2.49	0.01	0.00	0.03	0.16
	StimQ1	0.02	0.02	0.08	0.96	0.34	−0.02	0.05	0.07
SciLit3	Intercept	−1.62	0.92	0.02	−1.76	0.08	−3.43	0.19	
	HomeAct1	0.12	0.04	0.26	2.92	0.00	0.04	0.20	0.24
	Lens1	0.38	0.10	0.34	4.02	<0.001	0.19	0.57	0.32
	NIH-ECB1	0.01	0.01	0.15	1.30	0.19	0.00	0.02	0.10
	StimQ1	−0.01	0.02	−0.01	−0.51	0.61	−0.05	0.03	−0.05
SciLit4	Intercept	−2.65	1.07	0.06	−2.48	0.01	−4.75	−0.55	
	HomeAct1	0.06	0.05	0.13	1.30	0.19	−0.03	0.16	0.12
	Lens1	0.37	0.12	0.27	3.13	0.00	0.14	0.60	0.28
	NIH-ECB1	0.01	0.01	0.24	1.68	0.09	0.00	0.03	0.15
	StimQ1	0.01	0.02	0.04	0.44	0.66	−0.03	0.05	0.04
SciLit5	Intercept	−1.51	1.09	0.03	−1.39	0.17	−3.65	0.63	
	HomeAct1	0.07	0.05	0.12	1.45	0.15	−0.03	0.16	0.14
	Lens1	0.31	0.11	0.15	2.81	0.01	0.09	0.53	0.26
	NIH-ECB1	0.01	0.01	0.20	1.43	0.15	0.00	0.03	0.13
	StimQ1	−0.00	0.02	−0.06	−0.30	0.76	−0.05	0.039	−0.03

SciLit, Scientific Literacy; Lens, Lens on Science; HomeAct, Home Science Activities; NIH-ECB, NIH toolbox Early Childhood Cognition Battery; CI, confidence interval; StimQ, StimQ-Preschool; LL, lower limit; UL, upper limit; sr, semi-partial (part) correlation. The number following the task abbreviation is the measurement wave. ^†^Scientific Literacy at Wave 3, 4, and 5 is a standardized composite score but is a single score at Wave 1 and Wave 2.

In contrast, no predictive relations between access to science materials and subsequent scientific literacy hold when baseline scientific literacy is included as a control (all *ps* > 0.10). None of the few reciprocal correlations between early scientific literacy and later home science activities or materials observed in Table 10 hold in regression analyses when controlling for baseline levels of the corresponding aspect of the home science environment (all *ps* > 0.10).

### 3.3. How specific are observed relations between the home science environment and scientific literacy?

Given that there are many ways that children’s home environment might contribute to the development of their scientific literacy, it was important to distinguish our measure of the home science environment from the broader richness of the home environment. We therefore added the StimQ-P, the more general measure of the home learning environment, as another predictor into our regression models. While scores on the StimQ-P positively correlated with our measure of the home science environment at Wave 1 (*r = 0*.53, *p* < 0.001), it was not to the degree to cause a collinearity threat. The StimQ-P did not significantly predict scientific literacy at Wave 2, *B* = 0.02 (SE = 0.02), *p* = 0.409, nor Wave 3, *B* = −0.01 (SE = 0.02), *p* = 0.683. Home science environment (as well as the activities component alone) at Wave 1, on the other hand, continued to predict scientific literacy at Wave 3, *B* = 0.06 (SE = 0.03), *p* < 0.05, over and above StimQ-P (see Table 12). Because we did not collect StimQ-P scores at the second wave of measurement, we used Wave 1 scores as our closest approximation in regressions predicting scientific literacy from the Wave 2 home science environment. Here, the StimQ-P again failed to significantly predict scientific literacy at any wave, while the home science environment composite at Wave 2 did predict scientific literacy at Wave 5 (over and above StimQ-P), *B* = 0.06 (SE = 0.03), *p* < 0.05. In addition, excluding the home science environment factor entirely (i.e., leaving only StimQ-P in the regression model along with baseline scientific literacy) failed to yield any significant predictions to scientific literacy at any time point.

**Table 12:** Multivariate regression analysis for home science environment at Waves 1 and 2 predicting scientific literacy (with StimQ).

Outcome	Predictor	Unstandardized		Standardized	*t*	*p*	95% CI		*sr*
		*B*	SE	β			*LL*	*UL*	
**Wave 1**									
SciLit2	Intercept	−1.01	0.75	−0.07	−1.33	0.18	−2.48	0.47	
	HomeSci1	0.03	0.02	0.09	1.08	0.28	−0.02	0.07	0.08
	Lens 1	0.50	0.09	0.51	5.58	<0.001	0.32	0.67	0.41
	NIH-ECB1	0.01	0.01	0.21	2.57	0.01	0.00	0.03	0.16
	StimQ1	0.02	0.02	0.08	0.83	0.41	−0.02	0.05	0.07
SciLit3	Intercept	−1.65	0.94	0.01	−1.75	0.08	−3.49	0.20	
	HomeSci1	0.06	0.03	0.22	2.05	0.04	0.00	0.11	0.18
	Lens1	0.38	0.10	0.33	3.90	<0.001	0.19	0.57	0.32
	NIH-ECB1	0.01	0.01	0.18	1.51	0.13	0.00	0.02	0.12
	StimQ1	−0.01	0.02	−0.02	−0.41	0.68	−0.05	0.03	−0.04
**Wave 2**									
SciLit3	Intercept	−2.08	0.86	0.06	−2.43	<0.001	−3.58	−0.40	
	HomeSci2	0.02	0.03	0.09	0.76	0.48	−0.03	0.07	0.07
	Lens2	0.52	0.11	0.51	4.97	<0.001	0.31	0.73	0.43
	NIH-ECB2	0.01	0.01	0.13	1.59	0.13	0.00	0.02	0.12
	StimQ1	−0.01	0.02	−0.01	−0.36	0.72	−0.04	0.03	−0.03
SciLit4	Intercept	−2.89	0.99	0.10	−2.93	<0.001	−4.51	−0.95	
	HomeSci2	0.05	0.03	0.20	1.76	0.05	0.00	0.11	0.18
	Lens2	0.47	0.14	0.40	3.38	<0.001	0.20	0.74	0.35
	NIH-ECB2	0.01	0.01	0.15	1.35	0.17	−0.01	0.03	0.12
	StimQ1	0.00	0.02	0.00	−0.02	0.99	−0.04	0.04	0.00
SciLit5	Intercept	−1.53	1.02	0.07	−1.75	0.13	−3.60	−0.47	
	HomeSci2	0.06	0.03	0.17	2.02	0.04	0.00	0.11	0.20
	Lens2	0.50	0.13	0.47	3.98	<0.001	0.24	0.75	0.41
	NIH-ECB2	0.01	0.01	0.12	1.05	0.43	−0.01	0.02	0.09
	StimQ1	−0.02	0.02	−0.14	−1.01	0.31	−0.07	0.02	−0.11

SciLit, Scientific Literacy at Wave 3, 4, and 5 is a standardized composite score but is a single score at Wave 1 and Wave 2; Lens, Lens on Science; HomeSci, Home Science Environment; NIH-ECB, NIH Toolbox Early Childhood Cognition Battery; StimQ, StimQ-Preschool; CI, confidence interval; LL, lower limit; UL, upper limit; sr = semi-partial (part) correlation. The number following the task abbreviation is the measurement wave.

Lastly, we considered whether aspects of the home science environment specifically predict scientific literacy, as opposed to more generally predicting achievement in a domain-general way. To this end, we conducted a bivariate correlation analysis with measures of two conceptually distinct academic domains (the TerraNova Math subtest and the NIH Oral Reading Recognition task) taken at the last measurement time point. No associations between these outcomes and any aspect of early input (i.e., parent causal invitations, the home science environment composite, materials or activities) were detected (see Table 13). We therefore did not proceed with further regression analyses.

**Table 13:** Bivariate correlations for home science experience and math and literacy achievement.

	Home science environment									
	Wave 1		Wave 2		Wave 3		Wave 4		Wave 5	
	Act	Mat	Act	Mat	Act	Mat	Act	Mat	Act	Mat
Math	0.13	0.03	0.08	0.05	−0.05	0.01	0.04	0.01	0.14	0.23
Literacy	0.28	0.18	0.05	0.13	0.07	0.13	0.07	0.17	−0.11	0.27

Act, Activity; Mat, Material; Math, TerraNova Math Subtest taken at Wave 5; Literacy, NIH Toolbox Oral Reading Recognition Test taken at Wave 5.

## 4. Discussion

The goal of this longitudinal project was to broaden understanding of the relations between early science-related input and children’s emergent scientific literacy by building upon data first collected at 3 years of age and reported in Booth et al. (2020). In that initial analysis, the degree to which parents invited their children to explain causal phenomena, but not the degree to which they provided causal explanations themselves, correlated with children’s contemporaneous scientific literacy. Although it was only considered as a control variable in Booth et al. (2020), the home science environment also accounted for unique variance in children’s scientific literacy. In the current work, we therefore considered further parent invitations of causal explanations, while also closely examining the broader home science literacy environment. Overall, our investigation revealed that exposure to science-related input plays a particularly powerful role in shaping scientific literacy when children are very young and just entering preschool. This broad conclusion holds when considering both parent causal-explanatory talk and exposure to science-related home environment, but the latter effects were substantially more robust and long-lasting.

With respect to parent causal-explanatory talk, we found that parent invitations to explain causal phenomena were related to children’s scientific literacy concurrently and one year later. However, because this singular longitudinal association failed to hold when controlling for initial scientific literacy, no clearly predictive relations were evident. It is entirely possible that, if a predictive relation between parent invitations to explain and scientific literacy exists at all, it is confined to only the very earliest developmental window. Any correlations to later scientific literacy might well be due to subsequent stability in outcome measurements. Notably, no associations between the degree to which parents provided explanations themselves and children’s concurrent or subsequent scientific literacy were observed at all. Although surprising given the theoretically plausible usefulness of these explanations for building scientific knowledge, this finding is consistent with results reported for the first wave of data collection in Booth et al. (2020). It is also consistent with evidence suggesting that explanations might actually curtail learning under some circumstances by undermining children’s own exploration and discovery process (e.g., Bonawitz et al., 2011; Brockbank and Walker, 2022).

Together, these results suggest that parent causal-exploratory talk plays little role in shaping emergent scientific literacy. However, related research has found parent explanatory talk to be positively related to children’s concurrent science-related exploration and learning (Fender and Crowley, 2007; Marcus et al., 2018; Willard et al., 2019). Studies also find young children’s self-generated causal explanations to promote foundational scientific skills such as causal learning, and hypothesis generation, and revision (e.g., Walker et al., 2017; Busch et al., 2018).

Our failure to observe robust associations between aspects of parent causal talk and children’s scientific literacy might be due to limitations of our measurement approach. First, our observations were quite brief and constrained to laboratory settings (with the exception of the one museum observation at 3 years). While the play materials available in these contexts were intended to evoke natural conversations about causality, other or more varied options might have been more successful. More extended recordings in the home would have perhaps been most ideal for capturing natural variation. Second, our coding of the existing data did not capture potentially important nuances in the input. For example, we did not consider the quality of explanations produced by parents or children. Studies find explanations produced by parent and children are not always accurate or exhaustive (Snow and Kurland, 1996; Kelemen, 1999; Crowley et al., 2001; Gelman, 2003), and it might be that quality is an important moderator of relations between parent talk and children’s scientific literacy (Fender and Crowley, 2007; Mills et al., 2022). Parent invitations for children to explain might also be most likely to elicit responses when they are attuned to the child’s level of knowledge and interests (Chouinard et al., 2007). Finally, given that the only hints of association between parent causal-explanatory talk and children’s scientific literacy were evident at our earliest 3-year-old measurement, it is also possible that effects would have been stronger if we observed even earlier developmental windows.

In contrast to the relatively weak effects observed in consideration of parent causal-explanatory talk, our analyses of the broader home science environment were much more promising. Specifically, we found the home science environment at Wave 1 to be related to contemporaneous scientific literacy, as well as to scores at subsequent Waves 2 and 3. These longitudinal relations held even when controlling for initial scientific literacy scores and could not be fully accounted for by general cognitive abilities. Similarly, the home science environment measured at Wave 2 predicted concurrent scientific literacy, as well as scores at all subsequent measurement waves. These relations variably held when baseline scientific literacy and cognitive skills were included as controls. Importantly, reciprocal relations between early scientific literacy and later home science environment were rare and relatively weak, failing to maintain after controlling for baseline home science environment.

Interestingly, the most robust and longest lasting associations were observed in exploratory analyses differentiating home science activities from access to science-related materials. Indeed, home science activities, such as conducting experiments and visiting science museums, measured at 3 years as children were entering preschool, predicted scientific literacy through first grade. Home science materials, in contrast, failed to maintain any predictive power on their own (after controlling for baseline scientific literacy). One possibility is that materials are less important to emergent scientific literacy because children these young are unable to gain much from them of conceptual value through exploration on their own. Their value might therefore derive entirely from interactions they stimulate with parents, which would be subsumed by our measure of home science activities. It is also entirely possible that learning about the causal fundamentals of science at these early ages can be easily achieved with household items in the context of everyday activities like bathing and cooking, thus obviating the need for extensive collections of specifically science-themed materials.

Although nuanced, and in need of replication and further confirmation through intervention studies, the overall pattern of results is consistent with enduring effects of the home science environment on the subsequent development of children’s scientific literacy. Moreover, this relation appears to be quite specific. Effects of science-related aspects of the home environment were generally stronger than those of cognitive stimulation more generally speaking, and some of the former maintained even when controlling for the latter. In addition, the home science environment differentially predicted scientific, in contrast to math or reading, literacy.

In sum, this project demonstrates that children’s exposure to science related experiences as they enter preschool are predictive of their scientific literacy up to 4 years later. The fact that experiences at home appear to relatively decrease in importance as children spend more time in other contexts (e.g., preschool) is consistent with broad socio-ecological theories of development (e.g., Bronfenbrenner, 1986). Interestingly, a similar developmental pattern was observed for relations between children’s causal stance (i.e., interest and attunement to causality) and subsequent scientific literacy in another arm of this longitudinal project (Booth et al., 2022). In contrast, however, longer-lasting reciprocal relations were observed between children’s causal reasoning skills and developing scientific literacy (Shavlik et al., 2022). A better understanding of relations between these factors will be central to pinpointing the most impactful levers for addressing opportunity gaps and inequalities in science-related educational outcomes. Nevertheless, this study clearly converges on the importance of focusing on preschool, and potentially even earlier developmental windows as we proceed in these investigations.

## Data availability statement

The datasets presented in this study can be found in online repositories. The names of the repository/repositories and accession number(s) can be found at: OSF (https://osf.io/z7cgd).

## Ethics statement

The studies involving human participants were reviewed and approved by Institutional Review Board at Vanderbilt University, and Institutional Review Board at the University of Texas at Austin. Written informed consent to participate in this study was provided by the participants’ legal guardian/next of kin.

## Author contributions

AB and CH contributed to the design of the research. JB, MS, AB, and CH analysis of the results. JB, MS, CS, and AB wrote the manuscript. All authors contributed to the article and approved the submitted version.

## Funding

This work was graciously funded by the National Science Foundation (grant #1535102) awarded to AB and CH.

## Conflict of interest

The authors declare that the research was conducted in the absence of any commercial or financial relationships that could be construed as a potential conflict of interest.

## Publisher’s note

All claims expressed in this article are solely those of the authors and do not necessarily represent those of their affiliated organizations, or those of the publisher, the editors and the reviewers. Any product that may be evaluated in this article, or claim that may be made by its manufacturer, is not guaranteed or endorsed by the publisher.

## Footnotes

^1^ https://osf.io/y98g5

^2^ https://osf.io/b5mr8
